# Counterintuitive solvation effect of ionic-liquid/DMSO solvents on acidic C–H dissociation and insight into respective solvation†

**DOI:** 10.1039/c9sc06341b

**Published:** 2020-03-03

**Authors:** Wenzhi Luo, Chong Mao, Pengju Ji, Jun-Yan Wu, Jin-Dong Yang, Jin-Pei Cheng

**Affiliations:** Center of Basic Molecular Science (CBMS), Department of Chemistry, Tsinghua University Beijing 100084 China jdyang@mail.tsinghua.edu.cn jinpei_cheng@mail.tsinghua.edu.cn; State Key Laboratory of Elemento-organic Chemistry, College of Chemistry, Nankai University Tianjin 300071 China

## Abstract

How would acidic bond dissociation be affected by adding a small quantity of a weakly polar ionic liquid IL (the “apparent” or “measured” dielectric constant *ε* of the IL is around 10–15) into a strongly polar molecular solvent (*e.g.*, *ε* of DMSO: 46.5), or *vice versa*? The answer is blurred, because no previous investigation was reported in this regard. Toward this, we, taking various IL/DMSO mixtures as representatives, have thoroughly investigated the effects of the respective solvent in ionic–molecular binary systems on self-dissociation of C–H acid phenylmalononitrile PhCH(CN)_2_*via* p*K*_a_ determination. As disclosed, in this category of binary media, (1) no linear correspondence exists between p*K*_a_ and molar fractions of the respective solvent components; (2) only ∼1–2 mol% of weakly polar ILs in strongly polar DMSO make C–H bonds even *more* dissociative than in neat DMSO; (3) a small fraction of DMSO in ILs (<10 mol%) can dramatically ease acidic C–H-dissociation; and (4) while the DMSO fraction further increases, its acidifying effect becomes much attenuated. These findings, though maybe counterintuitive, have been rationalized on the basis of the precise p*K*_a_ measurement of this work in relation to the respective roles of each solvent component in solvation.

## Abstract 2

The dependence of PhCH(CN)_2_ p*K*_a_ on the molar fraction of ionic liquids in ionic–molecular binary mixtures showed a nonlinear three-fragment plot, which was rationalized for the first time by the respective roles of each solvent component for solvation.

## Graphical abstract

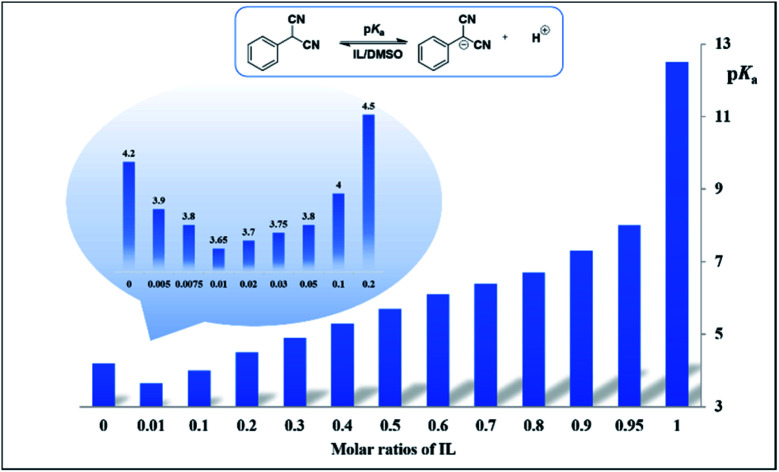

## Introduction

The primary difference between ionic liquids (ILs) and conventional media is that ILs contain entirely ions, while the counterpart has only molecules in composition. This, in fact, splits them into two conceptually distinct categories of medium systems with respect to the roles of ions *vs.* molecules in solvating solute to ease heterolytic bond-scission for reaction. As the field of ILs remains rapidly growing^1–5^ and the combined use of ILs with conventional solvents has become more and more popular through all these years,^6–8^ it is desirable to know some guiding principles regarding the power of solvation varied by the IL–molecular binary solvent, as compared to the situation for single or binary molecular media. However, except for some physical property studies of this category of binary media,^9–14^ sparse work could be found in the literature reporting their effect on facilitating a *chemical* process (where bonding situation is changed) such as acidic bond-dissociation.^15^

This is quite surprising, since among all kinds of chemical transformations, acidic R–H dissociation in conventional solvents,^16–20^ recently also in ionic media,^21–25^ often by means of p*K*_a_ study, is the most extensively investigated process, which not only laid the foundation for many most influential physical organic principles,^26–29^ but also provided some quantitative understandings of solvation effects on stabilizing various species generated upon heterolytic bond dissociation.

Among various IL–molecular dual-solvents, a mixture of imidazolium-based ILs and dimethyl sulfoxide (DMSO) is the most frequently used binary system.^30–34^ This is due to the superior solvation ability of DMSO towards organic compounds and its miscibility with most ILs. Hence, this combination of solvents was chosen as the working media in the present study to reveal the specific solvation behaviors of the IL–molecular binary system in general.

IL/DMSO mixtures have found many applications in organic chemistry such as dissolution and derivatization of cellulose and other biopolymers,^35–39^ Diels–Alder reactions,^40^ triazine synthesis,^41^ and so on. Structurally different ILs in such binary mixtures were found to have diverse promoting effects. It was noted that the composition ratio of mixtures is a crucial factor in affecting the dissolution and reactivity of reactants, and consequently in affecting the reaction outcomes.^42^ In this connection, improving a chemical process by tuning IL/DMSO mixtures would require quantitative knowledge of the solvation effects of these binary media varying with the changes of the respective molar ratios. However, as realized, there is essentially no detailed investigation on this respect in the literature. Such a situation urged us to carry out an in-depth investigation on the solvation effect of target binary solvents on heterolytic R–H bond dissociation in order to derive a useful quasi-quantitative guide for understanding their roles in solvation-assisted bond dissociation. In the present work, phenylmalononitrile was deliberately chosen as the model compound for probing the changes of its C–H p*K*_a_ in relation to the changes of solvent composition. The weak C–H bond of this molecule allows self-dissociation to occur and to be monitored by measuring the p*K*_a_ change (see Scheme 2) in a polar solvent without the need for any other species in the system, and therefore, perturbation likely brought in by other methodologies (*e.g.*, by using indicator overlapping or potentiometric titration) could be best minimized to ensure the high quality of the data.

As is well known, the dissociation constant (p*K*_a_) of a Brønsted acid is highly sensitive towards the structure and polarity of solvents. Therefore, it has well been used as an excellent probe to monitor the solvation effect in practice. Accordingly, several absolute p*K*_a_ scales in molecular solvents (*e.g.*, water,^43,44^ DMSO,^16–18^ acetonitrile ACN,^19,20^*etc.*) as well as in neat ILs^21–25^ were established in the past.^45,46^ Meanwhile, for some binary *molecular* solvent mixtures (*e.g.*, ACN/DMSO, H_2_O/EtOH, H_2_O/THF, *etc.*), the dependence of the acid–base equilibrium constants p*K*_a_ on the mixture compositions was also examined by some case studies, which generally exhibited a linear correspondence between the p*K*_a_ and the molar ratios.^47–51^ The lack of similar studies to examine the effect of solvation in IL–molecular mixtures made the chemical transformations occurring in these particular binary systems elusive and unpredictable, and in fact, hampered the rational development of the related areas. Toward this, we attempted to provide some insights into the solvation effect of IL–molecular mixtures on bond dissociation by monitoring the changes of the self-dissociation free energies (p*K*_a_) of the phenylmalononitrile C–H bond in four IL–molecular binary solvents of various molar combinations.

In the present work, the solvation effects of four IL/DMSO mixtures including [Bmim][NTf_2_]/DMSO, [Bmim][OTf]/DMSO, [Bmpy][NTf_2_]/DMSO and [DBUH][OTf]/DMSO were systematically examined by probing the variations of the acid self-dissociation constants p*K*_a_ of PhCH(CN)_2_ over the entire range of IL/DMSO compositions. The dependence of p*K*_a_ on mixture compositions exhibits three distinct regions, which are surprisingly different from the commonly observed linear correspondence in most molecular binary solvents.^47–51^ The effects of various IL–DMSO combinations on solvation-assisted acidic bond dissociation of the representative C–H acid were investigated and rationalized. The unexpected observations of this work actually provided a chance to find some insights into the distinct roles of each component in the IL–molecular system which should be useful for understanding the chemistry in IL/DMSO binary mixtures.

## Results and discussion

### Method of self-dissociation constant measurement

**Scheme 1:**
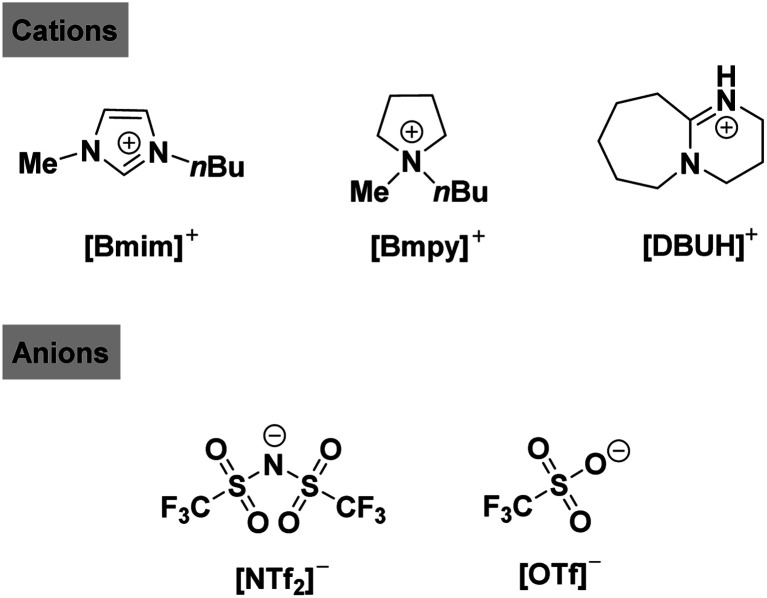
Structures of IL cations and anions used in the present work.

This group has established many p*K*_a_ scales for common or synthetically significant compounds previously in various aprotic/protic ILs^21–25^ and in DMSO^16–18^ using the familiar indicator overlapping method. However, to date, no absolute p*K*_a_ scales have been established in any IL–molecular binary medium. Here, we used a different approach (see the ESI† for details), which depends on monitoring the UV-vis absorption of the carbanion generated upon C–H bond self-dissociation of a suitable molecule in a series of binary media of DMSO and aprotic/protic ILs (Scheme 1), rather than on that of an external indicator, to investigate the bond dissociation free energy (*i.e.*, the absolute p*K*_a_).

**Scheme 2:**
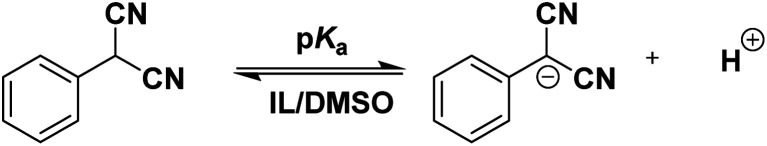
Self-dissociation of indicator acid PhCH(CN)_2_ in IL/DMSO mixtures.

In contrast to the conventional indicator overlapping method, known to be highly precise for p*K*_a_ determination but has to rely on the use of other indicator acids of known absolute acidity, the p*K*_a_ values in a binary IL/DMSO mixture were measured otherwise in an absolute sense, against the solvent as the standard reference, by monitoring the degree of R–H bond self-dissociation with a UV-vis spectrophotometer (*cf.* ESI†). The feasibility of the measurement requests at least two primary conditions: (1) the R–H bond of the targeted acid has to be weak enough to allow acidic self-dissociation to take place in the absence of any outside chemical except for the solvent, and (2) its conjugate base has to have a readily detectable UV-vis absorption which does not overlap with that of the parent acid. Once these basic requirements are satisfied, the self-dissociation equilibrium constant *K*_a_ can be directly determined and it provides p*K*_a_ upon a routine conversion. Then, finding such an acid capable of meeting all these prerequisites became a challenge. Besides the conditions, the most difficult part would actually be that this compound should allow self-dissociation to occur in the entire combination range of the binary solvent while still maintaining a dissociation–association equilibrium status. To our luck, we were finally able to find such a vital compound, PhCH(CN)_2_, that fulfills all the necessary requirements (Scheme 2). Its acidic dissociation constants, conventionally expressed as p*K*_a_, in four IL/DMSO binary mixtures were systematically determined over the whole component range *via* monitoring the amount of its conjugate carbanion PhC(CN)_2_^−^ by using a UV-vis spectrometer (see figures in the ESI†).

**Fig. 1:**
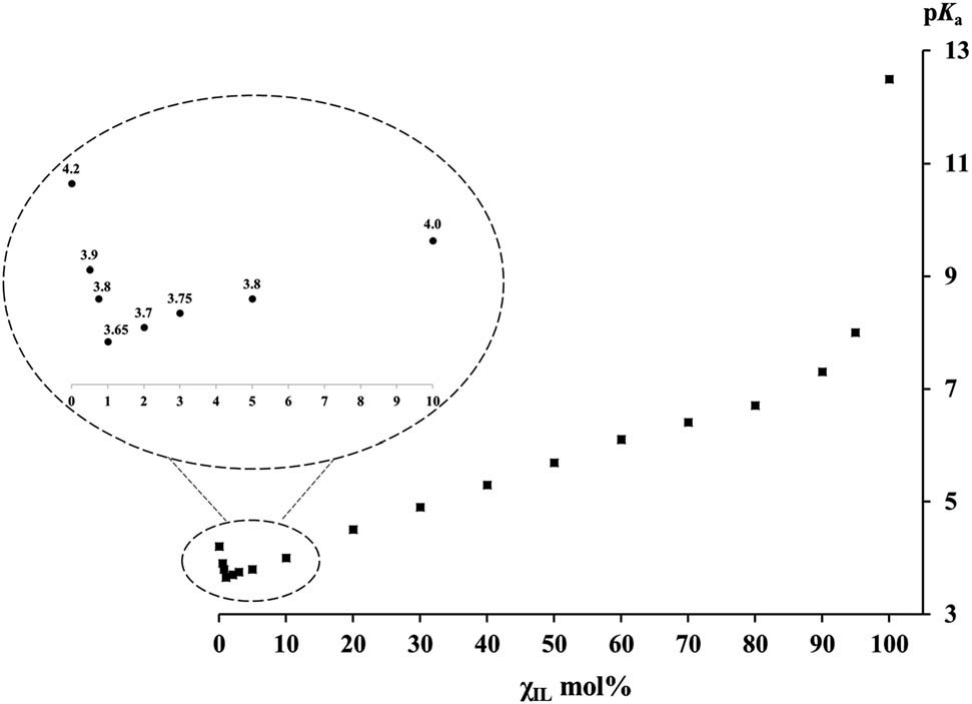
p*K*_a_ values of PhCH(CN)_2_*versus* the molar fractions *χ*_IL_ of [Bmim][NTf_2_] in [Bmim][NTf_2_]/DMSO mixtures.

The p*K*_a_ values of PhCH(CN)_2_ C–H bond self-dissociation in various IL/DMSO binary mixtures under standard conditions (*cf.* ESI†) are summarized in Table 1. The dependence of p*K*_a_ on the molar fractions of ILs (*χ*_IL_) is depicted in Fig. 1 (taking the [Bmim][NTf_2_]/DMSO mixture as an example). As mentioned, the most noteworthy advantages of this absolute acidity measurement are as follows: (1) this carbon acid and its conjugate base, *i.e.*, the carbanion, are least affected by specific effects such as ion-pairing, specific hydrogen-bonding, homoconjugation, *etc.*, so as to ensure the accurate determination of p*K*_a_ without perturbation of unwanted interactions, and (2) the UV-vis method gives equilibrium constants under true homogeneous conditions without the need for an outside heterogeneous surface (*e.g.*, electrode in the potentiostat) and supporting electrolyte, so the derived data are “true” values rather than “perturbed” values. By taking these advantages, both the experimental data and the insights disclosed from the data in this work should be reliable.

**Table 1:** p*K*_a_ values of PhCH(CN)_2_ in four IL/DMSO binary mixtures at various molar fractions of ILs *χ*_IL_ at 298 K^a^

*χ*_IL_ b (%)	p*K*_a_ of PhCH(CN)_2_^a^			
	[Bmim][NTf_2_]	[Bmim][OTf]	[Bmpy][NTf_2_]	[DBUH][OTf]
100	12.5	9.8	12.6	9.8
95	8.0	—	—	—
90	7.3	—	—	—
80	6.7	—	6.55^c^	6.25^c^
70	6.4	—	—	—
60	6.1	—	5.75^c^	5.35^c^
50	5.7	—	—	—
40	5.3	—	—	—
30	4.9	—	4.75^c^	4.4
20	4.5	—	—	—
10	4.0	3.9	4.0	3.75^c^
5	3.8	3.7	3.8	3.6
3	3.75^c^	3.7	3.75^c^	3.5
2	3.7	3.65^c^	3.7	3.4
1	3.65^c^	3.75	3.75^c^	3.6
0.75	3.8	—	—	—
0.5	3.9	3.8	3.8	3.75^c^
0	4.2	4.2	4.2	4.2

a In p*K*_a_ units; standard deviations ≤ ±0.05. The p*K*_a_ in neat DMSO (4.2)^52^ and in the four neat ILs^21^ is cited from the literature.

b *χ*_IL_, the molar fraction of the IL.

c The second decimal number for p*K*_a_.

### Distinct features of the p*K*_a_–*χ*_IL_ plot

In Fig. 1, the p*K*_a_–*χ*_IL_ plot for PhCH(CN)_2_ in [Bmim][NTf_2_]/DMSO mixtures shows three distinct regions, in sharp contrast to the situation reported in previous studies on molecular binary solvents where a linear correspondence was usually observed.^47–51^ The first thing noteworthy is that, in the bottom left region of Fig. 1 (also see the inset) where a varying trail of p*K*_a_ in DMSO with <10 mol% IL is presented, p*K*_a_ first goes down to a minimum (p*K*_a(min)_) value of 3.65 and then goes up. Note that this p*K*_a(min)_ of 3.65 in DMSO with only 1 mol% IL is even smaller than the p*K*_a_ of 4.2 in neat DMSO. It means that substitution of a very small amount of DMSO (∼1–2%) with a weakly polar IL can notably enhance the R–H bond acidic dissociation in the mixed solution. At the other end of Fig. 1, one can see a sudden p*K*_a_ drop from 12.5 (in the neat IL) to 8.0 with only 5 mol% DMSO added to the IL. In sharp contrast to the situation at the two ends, the middle part of the plot shows a basically linear response of p*K*_a_ to the variation of the IL/DMSO ratio, a phenomenon not too much different from those usually demonstrated by a molecular binary solvent. A similar p*K*_a_–*χ*_IL_ pattern was also found in the other three mixed IL–molecular solvent systems (*i.e.*, [Bmim][OTf], [Bmpy][NTf_2_] or [DBUH][OTf] with DMSO) (Table 1), indicating a general trend of IL/DMSO mixtures for acid dissociation by virtue of their cooperative roles in solvation.

### Solvation effect in IL–DMSO binary systems and insights disclosed

Generally, the dielectric constant (*ε*) could serve as a good index for identifying solvent polarity. While the use of this simple rule of thumb is quite successful in analyzing many solvent related problems encountered in conventional molecular solvents, it becomes complicated for ionic liquid chemistry.^53^ With this in mind, the analogous parameters of ILs may be viewed as “apparent” or “measured” polarity in relevant discussion in the present work. Since the apparent dielectric constants of most ILs fall in the range of 10 to 15,^54,55^ this category of media is usually regarded as weakly polar solvents if the criteria for molecular media are followed. On the other hand, the *ε* of 46.5 for DMSO^56^ indicates that its polarity is much stronger. In addition, the basicity of DMSO is also substantially greater than that of its counterpart IL in the IL/DMSO mixture, as judged by the proton solvation free energies (*e.g.*, Δ*G*_solv_(H^+^) is −270.3 and −252.8 kcal mol^−1^ in DMSO^57,58^ and [Bmim][NTf_2_],^59,60^ respectively). Both these two factors suggest that DMSO should be a much better acid-dissociating solvent than the IL. In fact, this is indeed verified by the PhCH(CN)_2_ p*K*_a_ values of 4.2 in DMSO^52^ and 12.5 in [Bmim][NTf_2_].^21^ In the same line of thinking, one would likely expect that, when an acid is dissolved in a binary solvent consisting of a weakly polar IL and the strongly polar DMSO, its acidity must be somewhere in between 4.2 of DMSO and 12.5 of [Bmim][NTf_2_], like what was commonly seen in a molecular bi-solvent. However, this is not the case in an IL/DMSO binary system, as exhibited by the data in Table 1 and the chart in Fig. 1. To understand this seemingly counterintuitive phenomenon, a tentative rationalization for it is proposed here below.

As one normally understood, the cation and anion of neat ILs are closely associated with each other in the form of intimate ion pairs (or clusters)^61^ due to strong coulombic interaction in between. When this IL is used as the working medium for p*K*_a_ measurement, the IL cation and anion could act as a whole in solvating the solutes in a way similar to the molecules of molecular media. From the basically linear responses of p*K*_a_*vs.* molar ratios of the IL/DMSO mixture in the mid-part of Fig. 1, one can expect that the IL may still remain largely as ion pairs when it is mixed with DMSO in the range of ∼10–90 mol%. Hence, it is understandable that the solvation behavior of the ionic and molecular solvent mixture in this region is not too much different from that of molecular bi-solvents because the intimate IL ion-pair may actually behave like a “molecule” under such circumstances.

However, there are numerous reports in the literature showing that ionic solutes, including liquidus salts like ILs, could also exist as free ions in both DMSO^16–18,62,63^ and ILs^21–23,64–66^ at low concentration. This reminds us to contemplate the roles of the IL cation and ion separately in solvating the solute in order to understand what is seen in the far left portion of Fig. 1 (also the inset). In this region where the molar concentration of the IL in DMSO is from 0 to 10%, the p*K*_a_ values of the probe C–H acid in the four IL/DMSO mixtures examined (Table 1) were all found to be smaller (*i.e.*, more acidic) than the value of 4.2 for PhCH(CN)_2_ in neat DMSO. This implies that the small quantity of the added IL must have played an even greater role in stabilizing PhC(CN)_2_^−^ and the proton than DMSO alone. One may first think that this would be impossible, because the apparent polarity and basicity of the IL^54,55^ have already been proved to be much weaker than those of DMSO,^56^ and if so, it leaves no way to enhance acidic bond dissociation. But, this was based on an assumption that the positive cation and the negative anion of the IL should always stay in pairs by intuition due to the internal electrostatic interaction. However, it has to be reminded that controversy over this assumption has never ceased. One may alternatively anticipate that, in dilute DMSO solution, the IL ion pairs could, at least in part, transform into free ions as already verified in the literature^64–66^ and in our previous studies as well.^6^ This would allow the IL cation to specifically interact with the carbanion upon C–H bond heterolysis. If this occurs, a better stabilization of the conjugate base of the acid could be realized because, compared to the weak solvating ability of DMSO towards organic anions, the IL cation (*e.g.*, [Bmim]^+^, [DBUH]^+^, *etc.*) is obviously better in stabilizing the acid anions. It is worth noting that there appears a critical point, regarding the IL fraction in DMSO, for maximizing the anion-stabilizing effect of the IL (*i.e.*, where p*K*_a(min)_ is measured), which is usually around ∼1–2 mol% of IL. When the IL concentration (*χ*_IL_) goes further beyond the critical point, its acidifying effect is attenuated presumably as a result of the regained ion-pair formation. For this, the IL effect could not be expected to be too large. Nevertheless, this acid-strengthening effect is already meaningful enough for consideration in the designs of some synthetic processes where ionic liquids are involved as additives or catalysts. The results presented here are basically consistent with the previously observed dependence of the sulfoxidation efficiency on the mole fraction of imidazolium ILs in IL/acetonitrile binary mixtures, where the maximum sulfoxide yield was reached at a molar fraction of 0.1–0.2.^67^ As mentioned above, this could be understood by considering the requisite change in ILs' self-aggregation in binary mixtures.^68,69^ Besides, the argument outlined above on the enhanced acidic dissociation of the substrate in molecular solvents with a small amount of IL added is also echoed with a kinetic investigation of methanolysis of alkyl chloride in [Bmim][NTf_2_]/MeOH mixtures, where a maximum rate was observed at an *χ*_IL_ of around 2 mol% IL.^70,71^ Their further kinetic study at varied temperature revealed that the addition of ILs to methanol is enthalpically beneficial but entropically unfavorable to the rate of the reaction. This may provide a clue for understanding the present observations from a different angle. Furthermore, it should also be pointed out that, although the small quantity of IL cation is most responsible for the further enhanced R–H acidity, the same amount of its counter anion could not much affect the acid dissociation because its amount is too small and the proton solvation energy by the NTf_2_^−^ anion is at least 10 kcal mol^−1^ weaker than that of DMSO (Δ*G*_solv_(H^+^) of −258.0 kcal mol^−1^ by NTf_2_^−^ ^59,60^*vs.* −270.3 kcal mol^−1^ by DMSO^57,58^).

Finally, when the IL content *χ*_IL_ further increases to be over 90 mol% (Fig. 1, far right), the p*K*_a_ value of PhCH(CN)_2_ shows a dramatic growth, *i.e.*, even with a thimbleful of DMSO added to the IL (*e.g.*, 1–5%), it could dramatically enhance the acidic dissociation of the acid. This is not unexpected, however, when considering the powerful solvation effect of DMSO on stabilizing the proton. Nevertheless, it is still quite surprising to see such a steep slope for this region compared to that of the preceding one. In this region, most IL cations and anions are in the form of intimate ion pairs. Consequently, PhC(CN)_2_^−^ should be primarily solvated by the residual dissociated IL cations, while the proton is mainly stabilized by the minor DMSO jointly with the IL. This points out a dramatic impact of trace adventitious additives (or may be viewed as “pollutants”) and reminds one that when IL is intended to be used as the standard medium of a reaction, great care has to be taken to ensure its high purity. Such a reminder could also be drawn from previous work where a significant impact of a trace of water on the hydrogen-bond donability of [Bmim][PF_6_]^72^ as well as on the reaction efficiency in [Bmim][NTf_2_] was demonstrated.^73^

In order to verify the generality of this synergetic solvation in IL–molecular mixtures, the other three IL/DMSO mixtures (aprotic [Bmim][OTf] and [Bmpy][NTf_2_], and protic [DBUH][OTf]) were further investigated. Similar results were obtained with p*K*_a(min)_ at *χ*_IL_ = 1–2 mol% in all the mixtures (see Table 1), although the exact values varied slightly (3.4–3.7). Considering that protons should be mainly solvated by DMSO rather than by the IL in the region around p*K*_a(min)_, it is reasonable to speculate that the small p*K*_a(min)_ variation in the four IL mixtures results primarily from the different solvation abilities of IL cations towards the PhC(CN)_2_^−^ anion.

Besides the C–H acid, the O–H self-dissociation characteristics of another probe acid 2,4-dinitrophenol have also been examined in two binary solvents ([Bmpy][NTf_2_]/DMSO and [DBUH][OTf]/DMSO) in an analogous manner (Table S1†). Again, it showed that a 1–2 mol% IL additive could reduce the p*K*_a_ of phenol from 5.1 in neat DMSO to a p*K*_a(min)_ of 4.6 in [Bmpy][NTf_2_]/DMSO and 4.5 in [DBUH][OTf]/DMSO. This indicates the generality of the synergetic solvation effect of the IL–molecular binary system.

## Conclusion

In the present work, the absolute p*K*_a_ scales of PhCH(CN)_2_ in four ionic–molecular binary systems over the entire composition range were established by precise determination of C–H bond self-dissociation free energies. Different from the linear responses usually observed in molecular binary mixtures, the dependence of p*K*_a_ on the molar fraction of ILs always showed a three-fragment plot as that in Fig. 1. The first section is the most surprising, that is, when a very small amount (∼1 mol%) of a weakly polar IL is added to the strongly polar DMSO, the dissociating power of the co-solvent becomes even stronger instead of weaker as anticipated. This is evident from the p*K*_a(min)_ of the probe acid under the mentioned conditions (3.65 *vs.* 4.2 in neat DMSO). The second part of the plot is normal, whereas the last section shows a sudden increase of p*K*_a_ with a steep slope when the molar fraction of the IL reaches 90% and above. These unexpected behaviors of the C–H bond acid-dissociation energies are rationalized for the first time for IL–molecular co-solvent systems on the basis of a detailed analysis of the respective roles of the IL, IL cation/anion, and molecular solvent in solvating the solutes and affecting the p*K*_a_ data.

## Conflicts of interest

There are no conflicts to declare.

## Supplementary Material

SC-011-C9SC06341B-s001
