# Supplementary material for: Counterintuitive solvation effect of ionic-liquid/DMSO solvents on acidic C–H dissociation and insight into respective solvation
Source: Chem Sci. 2020 Mar 3;11(12):3365–70. doi: 10.1039/c9sc06341b (PMC8152793; doi:10.1039/c9sc06341b)
Supplement: SC-011-C9SC06341B-s001 [file SC-011-C9SC06341B-s001.pdf]

Electronic Supplementary Material (ESI) for Chemical Science.
This journal is © The Royal Society of Chemistry 2020

Supporting Information for

Counterintuitive Solvation Effect of Ionic-Liquid/DMSO Solvents on Acidic C-H Dissociation and Insight into Respective Solvation

Wenzhi Luo,[a] Chong Mao,[a] Pengju Ji,[a] Jun-Yan Wu,[a] Jin-Dong Yang*[a] and Jin-Pei Cheng*[ab]

[a]*Center of Basic Molecular Science (CBMS), Department of Chemistry, Tsinghua University, Beijing, 100084, China.*

[b]*State Key Laboratory of Elemento-organic Chemistry, College of Chemistry, Nankai University, Tianjin, 300071, China.*

Contents

1. General Remarks......S2
2. Representative UV-vis spectra for pK_a measurements S3
3. pK_a values of 2,4-Dinitrophenol in IL/DMSO mixtures S12
4. References S12

1. General Remarks

Materials. The solvents used in the syntheses and measurements were purified according to *Solvent Purified Handbook*. Ionic liquids and indicator acids were synthesized and purified based on literature procedures.[1] The solutions of the indicator acids (phenylmalononitrile and 2,4-Dinitrophenol), and base (1,1-bis(ethylsulfonyl)-2-methylpropan-1-ide) were prepared as that described in our previous work.[2-6] The water contents were less than 50 ppm for ILs and 10 ppm for DMSO, which were determined by the Karl-Fischer method. UV spectra were obtained on a Hitachi U-3900H UV-Vis spectrophotometer.

pK_a Measurement in Mixed Solvents. All the preparation and measurement processes were performed under water- and oxygen-free conditions. Standard Schlenk techniques were applied throughout the whole manipulation under dry argon atmosphere. pK_as of indicator acids in the mixtures with the molar ratios of IL/DMSO varying from 0 to 1 were measured by determining the equilibrium position of their self-dissociation on UV spectrometer. ***Step 1***, the molar absorptivity (ε) of the indicator anion was measured through the titration of a base solution with an indicator solution, where the specific absorption peaks of indicator anions (about 300 nm for phenylmalononitrile anion, and 430 nm for 2,4-dinitrophenol anion) were chosen as the detecting signal (See chemical equation S1 and eq. S1). ***Step 2***, indicator acids were added dropwise to blank IL/DMSO mixtures, and the concentration of the dissociated anion for each equilibrium can be calculated from the UV absorption and the molar absorptivity determined previously. Then, combined with the total amount of the indicator acid used, the dissociation degree and pK_a value of this acid in the present mixture can be derived (See chemical equilibrium S2 and eq. S2).

Taking Figure S1 as an example. ***Step 1***, The molar absorptivity (ε) of the indicator anion in this mixed solvent is equal to the slope of the plot which related several sets of anion concentrations c(In^-) with corresponding absorbances $A_b(In^-)$ (See Figure S1a and c); ***Step 2***, pK_a value of each drop listed in Figure S1b and d can be calculated from the amount of the indicator acid c(HIn), the absorbance of the indicator anion $A_b(In^-)$ and the obtained molar absorptivity (ε) in *step 1* according to eq. S2.

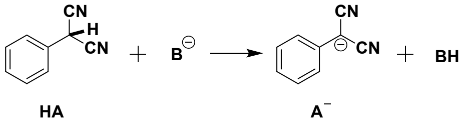

S1)

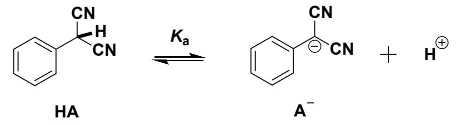

S2)

$A_b(In^-) = \varepsilon(In^-)*c(In^-)$ **eq. S1**

$$pK_a(HIn) = -\,lg\frac{c(In^-)\cdot c(H^+)}{c(HIn)} = -\,lg\frac{A_b(In^-)^2}{\varepsilon(In^-)^2\cdot c(HIn)}$$

eq. S2

Each pK_a value was obtained as the average of eight to ten titrations within each run, and the final pK_a was the average of two to three independent runs (SD ≤ 0.05 pK unit). In each experiment, the concentration of the indicator acid was controlled on the order of magnitude of 10^{-4} mol L^{-1} to avoid potential ion-pairing effect in solution.

2. Representative UV-vis spectra for pK_a measurements

All the measuring processes, from preparation of the indicator reservoir to the determination, were performed under strict water- and oxygen-free conditions. Thus, the custom-made UV cuvette and syringe were used (as shown below).

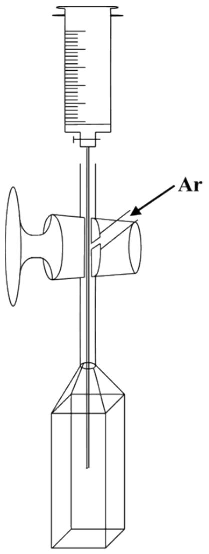

Representative spectra recorded during the pK_a measurement are given below. Others were obtained in similar procedures. The UV-vis spectra of indicator acids in IL/DMSO mixtures and experimental data are presented in Figures S1-S8.

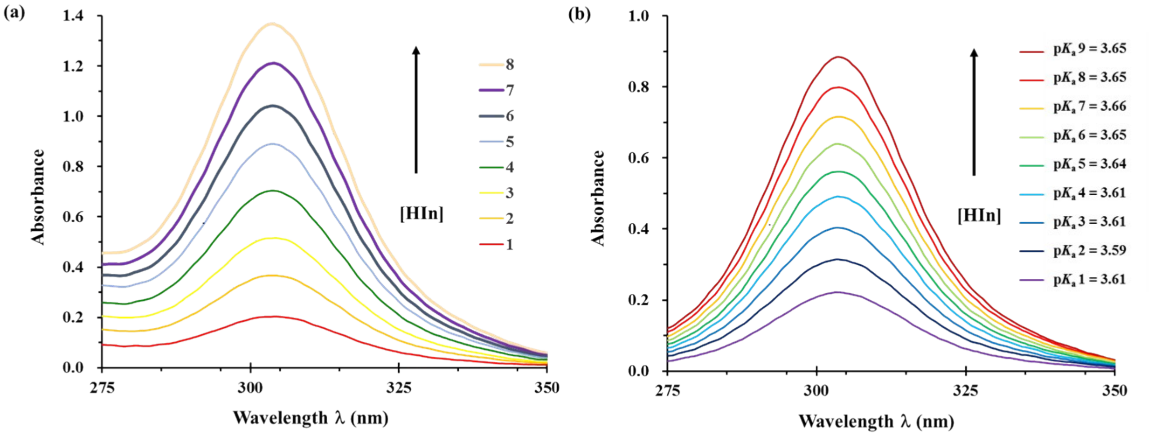

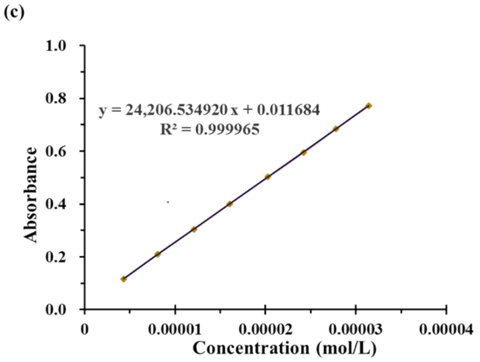

(d)

drop	(a)		(b)		
	$c_{HIn}/10^{-5}$M	absorbance	$c_{HIn}/10^{-5}$M	absorbance	pK_a
1	0.435	0.116	0.951	0.222	3.61
2	0.810	0.21	1.362	0.314	3.59
3	1.207	0.304	1.782	0.404	3.61
4	1.607	0.401	2.196	0.491	3.61
5	2.028	0.502	2.552	0.561	3.64
6	2.418	0.595	2.954	0.64	3.65
7	2.777	0.684	3.355	0.716	3.66
8	3.138	0.773	3.778	0.798	3.65
9			4.254	0.884	3.65

Figure S1. (a) UV-vis spectra of phenylmalononitrile anion when titrating the phenylmalononitrile solution into the base 1,1-bis(ethylsulfonyl)-2-methylpropan-1-ide in the mixture of 99 mol% DMSO and 1 mol% [Bmim][NTf_2]; **(b)** self-dissociation of phenylmalononitrile after each titration; **(c)** plot of the absorbance of phenylmalononitrile anion in figure **a** versus its concentrations for derivation of the molar absorptivity (ε); **(d)** experimental data for **a** and **b**.

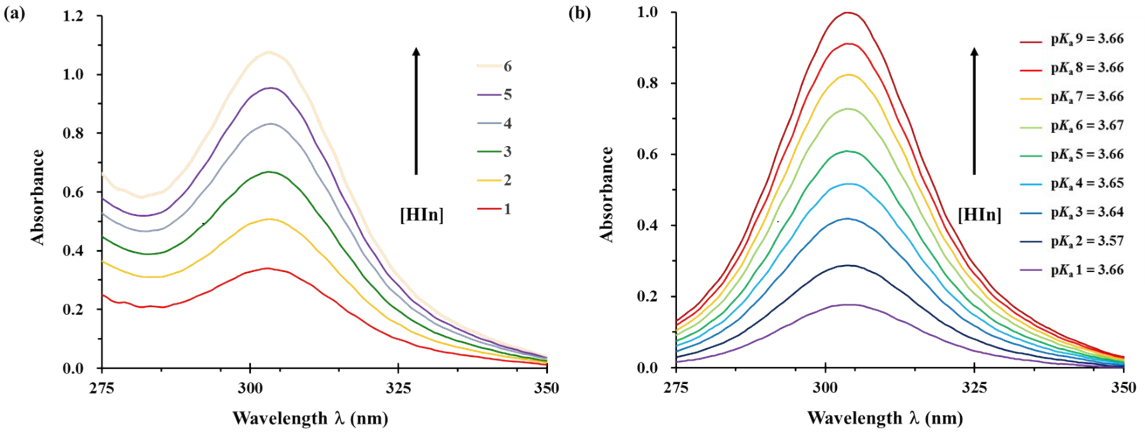

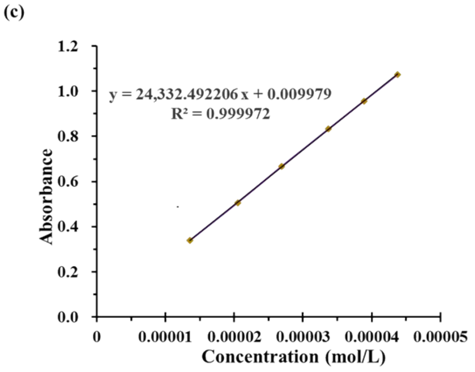

(d)

drop	(a)		(b)		
	c_{HIn}/10^{-5}M	absorbance	c_{HIn}/10^{-5}M	absorbance	pK_a
1	1.357	0.340	0.752	0.177	3.66
2	2.049	0.507	1.231	0.287	3.57
3	2.692	0.667	1.846	0.418	3.64
4	3.372	0.832	2.323	0.516	3.65
5	3.887	0.956	2.788	0.609	3.66
6	4.379	1.074	3.406	0.727	3.67
7			3.904	0.822	3.66
8			4.389	0.912	3.66
9			4.886	0.999	3.66

Figure S2. (a) UV-vis spectra of phenylmalononitrile anion when titrating the phenylmalononitrile solution into the base 1,1-bis(ethylsulfonyl)-2-methylpropan-1-ide in the mixture of 98 mol% DMSO and 2 mol% [Bmim][NTf_2]; **(b)** self-dissociation of phenylmalononitrile after each titration; **(c)** plot of the absorbance of phenylmalononitrile anion in figure **a** versus its concentrations for derivation of the molar absorptivity (ε); **(d)** experimental data for **a** and **b**.

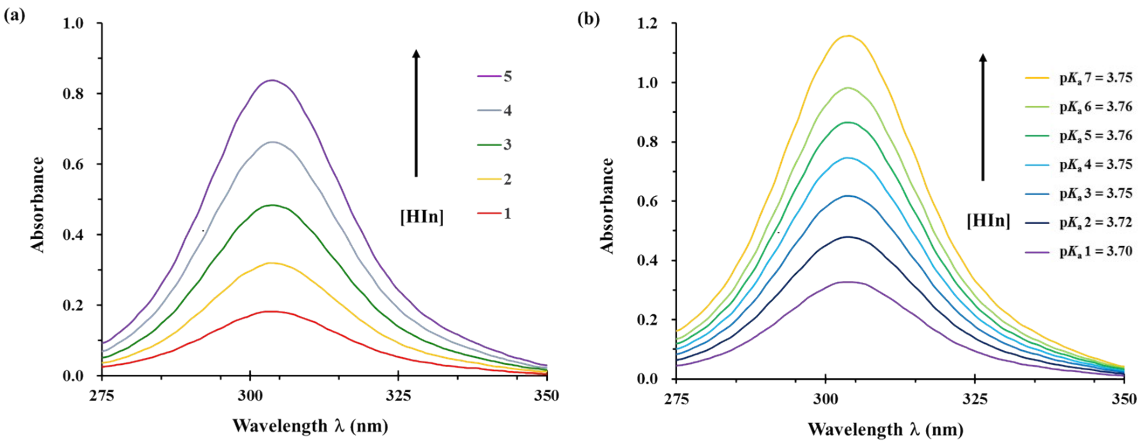

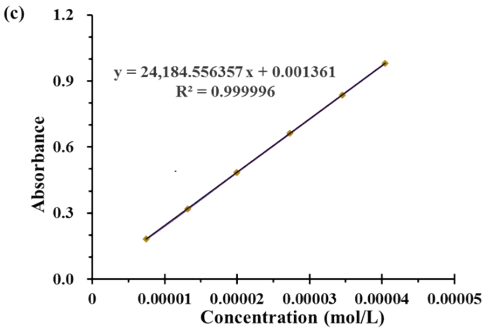

(d)

drop	(a)		(b)		
	$c_{HIn}/10^{-5}$M	absorbance	$c_{HIn}/10^{-5}$M	absorbance	pK_a
1	0.744	0.182	1.442	0.327	3.69
2	1.322	0.320	2.191	0.480	3.72
3	1.993	0.484	2.923	0.618	3.75
4	2.732	0.662	3.630	0.747	3.75
5	3.455	0.837	4.330	0.865	3.76
6	4.0427	0.979	5.012	0.980	3.76
7			6.068	1.154	3.75

Figure S3. (a) UV-vis spectra of phenylmalononitrile anion when titrating the phenylmalononitrile solution into the base 1,1-bis(ethylsulfonyl)-2-methylpropan-1-ide in the mixture of 98 mol% DMSO and 2 mol% [Bmpy][NTf_2]; **(b)** self-dissociation of phenylmalononitrile after each titration; **(c)** plot of the absorbance of phenylmalononitrile anion in figure **a** versus its concentrations for derivation of the molar absorptivity (ε); **(d)** experimental data for **a** and **b**.

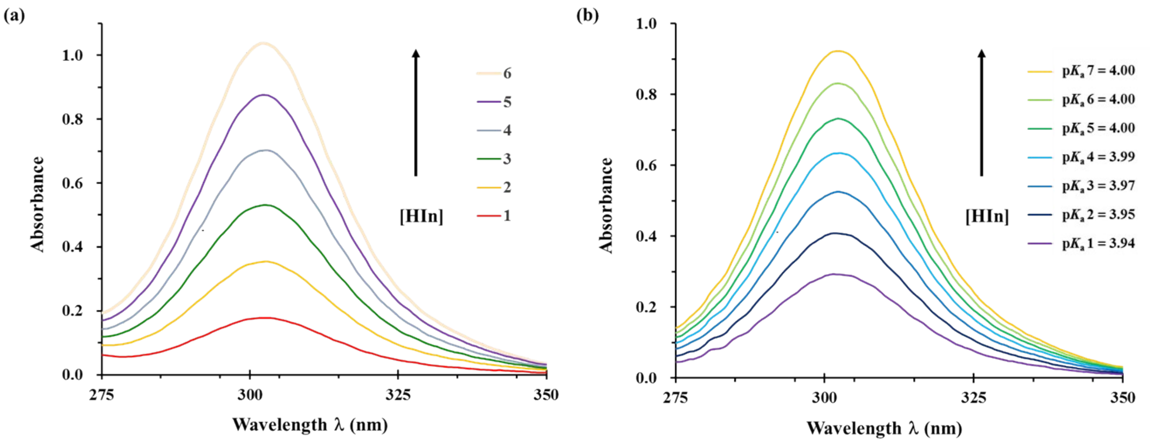

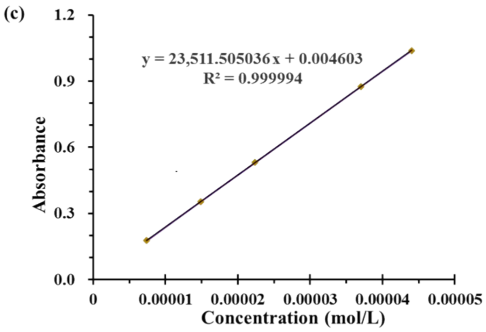

(d)

drop	(a)		(b)		
	$c_{HIn}/10^{-5}$M	absorbance	$c_{HIn}/10^{-5}$M	absorbance	pK_a
1	0.737	0.177	1.375	0.292	3.94
2	1.483	0.354	1.999	0.407	3.95
3	2.237	0.531	2.693	0.525	3.97
4	3.702	0.876	3.412	0.634	3.99
5	4.404	1.039	4.079	0.732	4.00
6			4.763	0.830	4.00
7			5.454	0.922	4.00

Figure S4. (a) UV-vis spectra of phenylmalononitrile anion when titrating the phenylmalononitrile solution into the base 1,1-bis(ethylsulfonyl)-2-methylpropan-1-ide in the mixture of 90 mol% DMSO and 10 mol% [Bmpy][NTf_2]; **(b)** self-dissociation of phenylmalononitrile after each titration; **(c)** plot of the absorbance of phenylmalononitrile anion in figure **a** versus its concentrations for derivation of the molar absorptivity (ε); **(d)** experimental data for **a** and **b**.

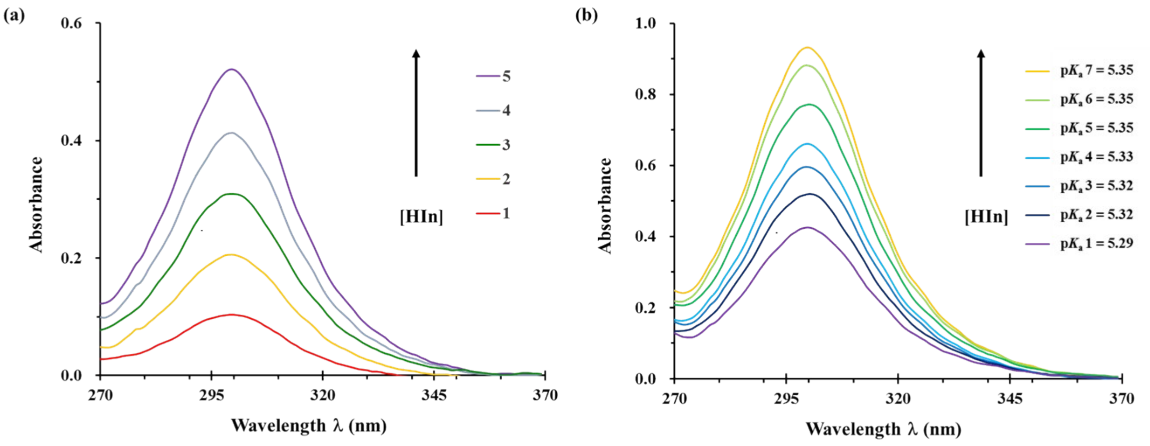

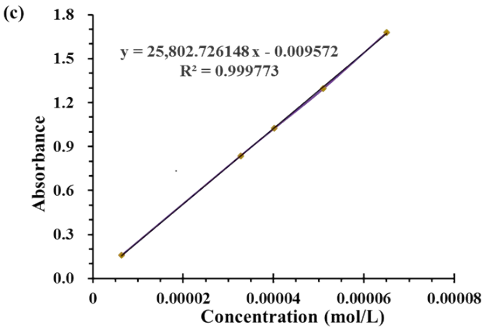

(d)

drop	(a)		(b)		
	$c_{HIn}/10^{-5}$M	absorbance	$c_{HIn}/10^{-5}$M	absorbance	pK_a
1	0.633	0.157	9.695	0.426	5.29
2	3.272	0.837	14.827	0.519	5.32
3	4.019	1.024	18.853	0.595	5.32
4	5.104	1.295	23.233	0.660	5.33
5	6.508	1.680	32.965	0.773	5.35
6			42.631	0.881	5.35
7			47.127	0.932	5.35

Figure S5. (a) UV-vis spectra of phenylmalononitrile anion when titrating the phenylmalononitrile solution into the base 1,1-bis(ethylsulfonyl)-2-methylpropan-1-ide in the mixture of 40 mol% DMSO and 60 mol% [DBUH][OTf]; **(b)** self-dissociation of phenylmalononitrile after each titration; **(c)** plot of the absorbance of phenylmalononitrile anion in figure **a** versus its concentrations for derivation of the molar absorptivity (ε); **(d)** experimental data for **a** and **b**.

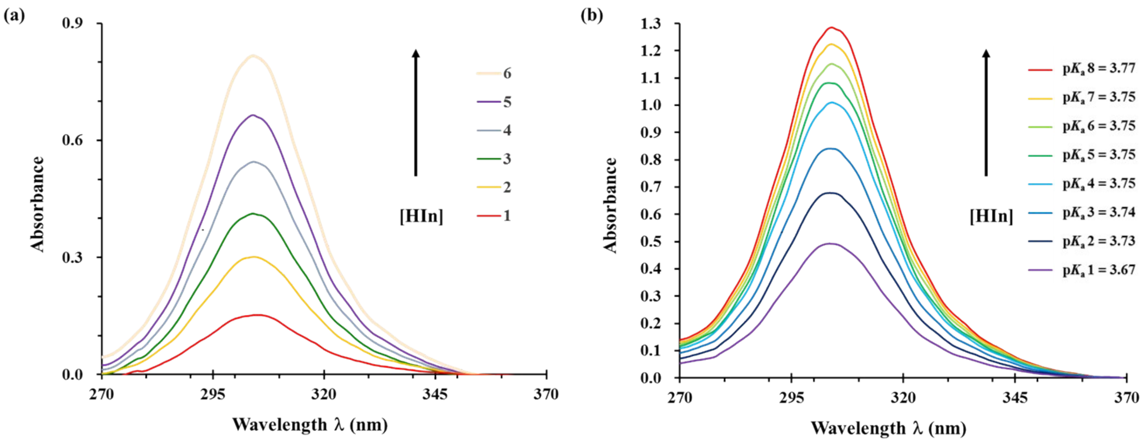

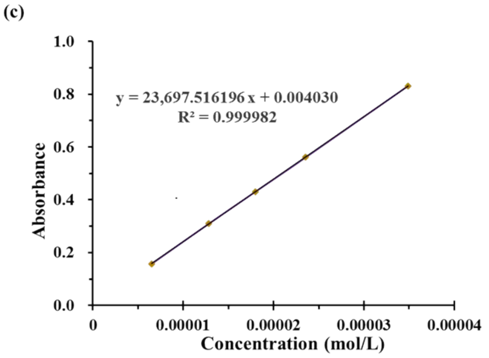

(d)

drop	(a)		(b)		
	$c_{HIn}/10^{-5}$M	absorbance	$c_{HIn}/10^{-5}$M	absorbance	pK_a
1	0.6529	0.158	2.285	0.493	3.67
2	1.285	0.310	3.294	0.678	3.73
3	1.796	0.430	4.228	0.840	3.74
4	2.356	0.561	5.289	1.012	3.75
5	3.488	0.831	5.734	1.081	3.75
6			6.205	1.152	3.75
7			6.686	1.226	3.75
8			7.159	1.286	3.77

Figure S6. (a) UV-vis spectra of phenylmalononitrile anion when titrating the phenylmalononitrile solution into the base 1,1-bis(ethylsulfonyl)-2-methylpropan-1-ide in the mixture of 99.5 mol% DMSO and 0.5 mol% [DBUH][OTf]; **(b)** self-dissociation of phenylmalononitrile after each titration; **(c)** plot of the absorbance of phenylmalononitrile anion in figure **a** versus its concentrations for derivation of the molar absorptivity (ε); **(d)** experimental data for **a** and **b**.

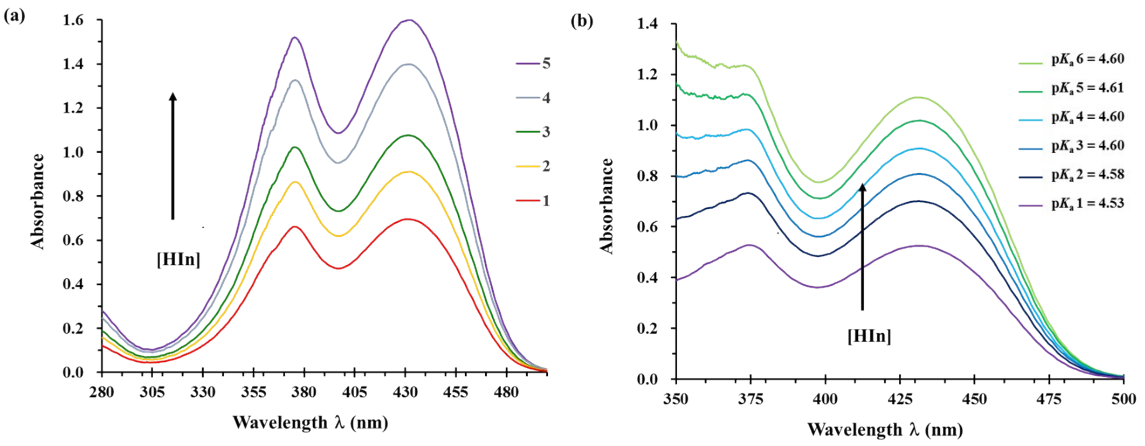

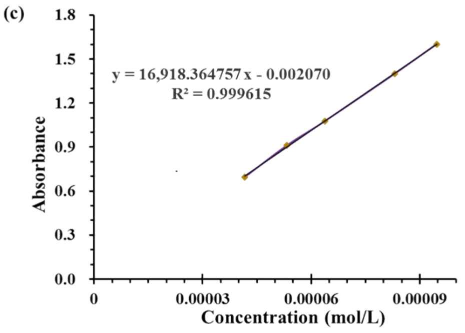

(d)

drop	(a)		(b)		
	$c_{HIn}/10^{-5}$M	absorbance	$c_{HIn}/10^{-5}$M	absorbance	pK_a
1	4.169	0.696	6.389	0.525	4.53
2	5.330	0.911	10.689	0.701	4.58
3	6.377	1.076	13.789	0.808	4.60
4	8.309	1.399	16.789	0.909	4.60
5	9.466	1.601	20.389	1.018	4.60
6			23.589	1.109	4.60

Figure S7. (a) UV-vis spectra of 2,4-dinitrophenol anion when titrating the 2,4-dinitrophenol solution into the base 1,1-bis(ethylsulfonyl)-2-methylpropan-1-ide in the mixture of 98 mol% DMSO and 2 mol% [DBUH][OTf]; **(b)** self-dissociation of 2,4-dinitrophenol after each titration; **(c)** plot of the absorbance of 2,4-dinitrophenol anion in figure **a** versus its concentrations for derivation of the molar absorptivity (ε); **(d)** experimental data for **a** and **b**.

(a) (b)

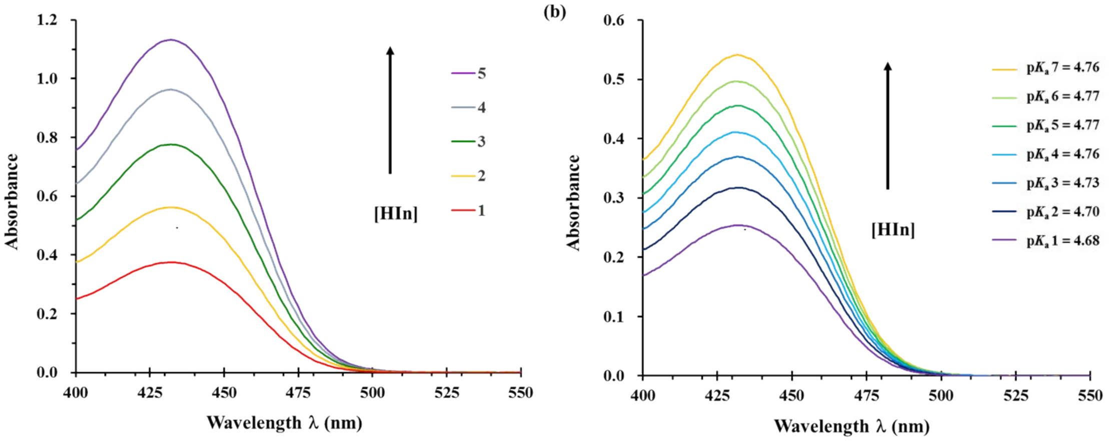

(c)

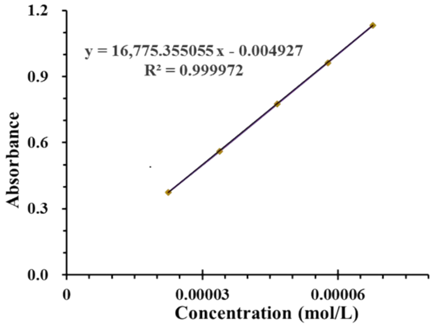

(d)

drop	(a)		(b)		
	$c_{HIn}/10^{-5}$M	absorbance	$c_{HIn}/10^{-5}$M	absorbance	pK_a
1	2.253	0.375	2.615	0.254	4.68
2	3.384	0.561	3.685	0.317	4.70
3	4.662	0.776	4.806	0.370	4.73
4	5.774	0.963	5.937	0.411	4.76
5	6.775	1.133	7.078	0.456	4.77
6			8.120	0.497	4.77
7			9.219	0.541	4.76

Figure S8. (a) UV-vis spectra of 2,4-dinitrophenol anion when titrating the 2,4-dinitrophenol solution into the base 1,1-bis(ethylsulfonyl)-2-methylpropan-1-ide in the mixture of 95 mol% DMSO and 5 mol% [Bmpy][NTf_2]; **(b)** self-dissociation of 2,4-dinitrophenol after each titration; **(c)** plot of the absorbance of 2,4-dinitrophenol anion in figure **a** versus its concentrations for derivation of the molar absorptivity (ε); **(d)** experimental data for **a** and **b**.

3. pK_a values of 2,4-Dinitrophenol in IL/DMSO mixtures

Table S1. pK_a values of 2,4-Dinitrophenol in binary IL/DMSO mixtures

	pK_a of 2,4-Dinitrophenol[b]	
$\boldsymbol{x_{IL}}$**(%)**[a]	**[Bmpy][NTf$_2$]**	**[DBUH][OTf]**
100	12.8	9
5	4.75	--
3		4.6
2	4.6	4.6
1	4.7	4.5
0	5.1	

[a]x_{IL}, molar fraction of IL in DMSO. [b]In pK units; standard deviations: ≤ ±0.05 pK units.

4. References

1 A. K. Burrell, R. E. D. Sesto, S. N. Baker, T. M. McCleskey, G. A. Baker, *Green Chem.*, 2007, **9**, 449–454.

2 H. Deng, X. Li, Y. Chu, J. He, J.-P. Cheng, *J. Org. Chem.*, 2012, **77**, 7291–7298.

3 Z. Wang, H. Deng, X. Li, P. Ji, J.-P. Cheng, *J. Org. Chem.*, 2013, **78**, 12487–12493.

4 Z. Wang, P. Ji, X. Li, J.-P. Cheng, *Org. Lett.*, 2014, **16**, 5744–5747.

5 C. Mao, Z. Wang, P. Ji, J.-P. Cheng, *J. Org. Chem.*, 2015, **80**, 8384–8389.

6 C. Mao, Z. Wang, Z. Wang, P. Ji, J.-P. Cheng, *J. Am. Chem. Soc.*, 2016, **138**, 5523–5526.
